# Quantifying the Beauty of Words: A Neurocognitive Poetics Perspective

**DOI:** 10.3389/fnhum.2017.00622

**Published:** 2017-12-19

**Authors:** Arthur M. Jacobs

**Affiliations:** ^1^Department of Experimental and Neurocognitive Psychology, Freie Universität Berlin, Germany; ^2^Dahlem Institute for Neuroimaging of Emotion, Berlin, Germany; ^3^Center for Cognitive Neuroscience Berlin, Berlin, Germany

**Keywords:** neurocognitive poetics, quantitative narrative analysis, machine learning, digital humanities, neuroaesthetics, computational stylistics, literary reading, decision trees

## Abstract

In this paper I would like to pave the ground for future studies in Computational Stylistics and (Neuro-)Cognitive Poetics by describing procedures for predicting the subjective beauty of words. A set of eight tentative word features is computed via Quantitative Narrative Analysis (QNA) and a novel metric for quantifying word beauty, the *aesthetic potential* is proposed. Application of machine learning algorithms fed with this QNA data shows that a classifier of the decision tree family excellently learns to split words into beautiful vs. ugly ones. The results shed light on surface and semantic features theoretically relevant for affective-aesthetic processes in literary reading and generate quantitative predictions for neuroaesthetic studies of verbal materials.

## The neurocognitive poetics perspective

When a reader's brain processes information about single words like “LOVELY” or “SHRIEK,” many neural circuits work together to enable meaning making. So far, practically all theoretical models have highlighted the neurocognitive processes underlying word recognition while neglecting the *affective-aesthetic* ones (for review: Hofmann and Jacobs, 2014; Jacobs et al., 2015). However, there is now abundant evidence that word recognition involves affective components from the first 100 ms of processing on (Kissler et al., 2007; Hofmann et al., 2009; for review see Citron, 2012). But there is practically no experimental research on aesthetic processes at the single word level (for exceptions, see Ponz et al., 2013; Jacobs et al., 2015). This is quite astonishing, given the success of neuroaesthetic research in other fields (e.g., Jacobsen et al., 2004; Jacobsen, 2006; Brattico et al., 2013; Leder, 2013; Nadal, 2013; Zeki et al., 2014; Marin, 2015) and work on the beauty of larger verbal materials, such as metaphors (McQuire et al., 2017), proverbs (Bohrn et al., 2013), idioms (Citron et al., 2016), or poems (Lüdtke et al., 2014; Hanauer, 2015).

The emerging field of Neurocognitive Poetics (Jacobs, 2015b,c; Willems and Jacobs, 2016) emphasizes such aesthetic processes during the reading of verbal materials in more natural and ecologically valid tasks and contexts and provides methods, e.g., QNA tools like the Berlin Affective Wordlist/BAWL (Võ et al., 2006, 2009), the DENN-BAWL (Briesemeister et al., 2011), EMOPHON (Aryani et al., 2013) or the Affective Norms for German Sentiment Terms/ANGST (Schmidtke et al., 2014a), as well as models for this field (e.g., the Neurocognitive Poetics Model/NCPM; Jacobs, 2015a; Jacobs and Willems, 2017; Nicklas and Jacobs, 2017).

The methodological challenge for this perspective is immense given the complexity of the verbal materials and the focus on processes that recruit more than the usual language circuits in the brain (e.g., Keidel et al., 2013; Jacobs and Willems, 2017). However, recent developments in QNA methods and machine learning, as well as in fMRI data analyses promise rapid progress in this regard. Thus, applications of QNA-based machine learning tools have allowed successful prediction of the liking of single words (Jacobs et al., 2016), classification of Shakespeare's 154 sonnets into motif categories (Jacobs et al., 2017), as well as predicting authorship, literariness and aptness of poetic metaphors (Jacobs and Kinder, 2017, in press), or subjective immersion into narratives (Jacobs and Lüdtke, 2017).

In this paper I show an application of such tools to predict the beauty/ugliness of single words from the Neurocognitive Poetics perspective in an attempt to motivate and generate more neuroscientific research on this issue.

## Micropoetry: the beauty of words and the origins of ludic reading

Beauty is an important human category, listed among the top features for almost all domains of aesthetic appreciation (Jacobsen and Beudt, 2017), the most prototypical aesthetic judgment (Jacobsen et al., 2004), and the most frequently used term for literature and poetry (Knoop et al., 2016). Readers often report the self-rewarding experience of beauty and harmony not only for entire poems (Jacobs, 2015b), but even for single words. This is documented in reports from the annual election of the most beautiful German word (Limbach, 2004). These examples show that words can be positive or negative, beautiful or ugly, and support the notion of *one-word poetry*, i.e., that single utterances or words—even outside lyrical contexts—can fulfill Jakobson's *poetic function* (Jakobson, 1960; Jacobs, 2015c; Jacobs and Kinder, 2015). However, there seems to be a single study so far that provides rating data on the beauty of single words, in German (Jacobs et al., 2015), while neuroimaging studies on that issue still are missing.

Understanding the neurocognitive bases of subjective feelings of the beauty of words and of micropoetic episodes is important for the investigation of more general and complex questions such as how language and emotion co-develop (Sylvester et al., 2016), how human beings come to like fiction (Jacobs and Willems, 2017), or how they acquire a taste for ludic reading and something like a lyrical sense (Jacobs and Kinder, 2015). Cognitive neuroscience so far has not even begun to shed light on the neural bases of the development of literary experiences (Jacobs, 2015c), although studies investigating the neural underpinnings of written language processing in children and adolescents are informative for the present purposes (e.g., Liebig et al., 2017).

## Predicting the beauty of words

In the behavioral study reported by Jacobs et al. (2015) standard linear (stepwise) regression analyses suggested that word beauty was best predicted by valence and familiarity *ratings* (*R*^2^lin = 0.77; AICc = 608), while the other two considered features, arousal and imageability, did not account for a significant part of variance in the beauty ratings for that sample. Note that these predictors were themselves based on ratings and thus on “subjective” measures. The most beautiful word was LIBELLE (dragonfly) with a mean rating of 6.1/7, followed by MORGENRÖTE (aurora, 5.9), and MITTSOMMERNACHT (midsummernight, 5.8). An additional hierarchical cluster analysis suggested that the most beautiful words described nine phenomena from nature (animals, flowers, rainbow etc.) and four states/objects of wellness (e.g., coziness), all rated high on beauty, valence, and imageability, and low on arousal. In contrast, the overall 24 ugliest words were almost all swear words associated with genitalia (see Jacobs et al., 2015, Supplementary Materials).

The multilevel hypothesis derived from the NCPM predicts that the liking of words, idioms, proverbs, sentences or entire poems is affected by nonlinear dynamic interactions of multiple features (or predictors) at multiple text levels, for example sublexical phonological features like phoneme salience with supralexical features like the global affective meaning (cf. Aryani et al., 2016). Powerful *decision tree* classifiers, e.g., extremely random trees/ERT which are the most accurate and efficient ones (Geurts et al., 2006) provide information about the importance of predictors from a large set (e.g., about 100; Jacobs et al., 2017), whether they are factorial or continuous, and even when there are more predictors than observations. They also work for unbalanced designs with high multicollinearity for which linear models are less appropriate (cf. Strobl et al., 2009; Tagliamonte and Baayen, 2012).

In contrast to Jacobs et al. (2015), here I exclusively used a set of QNA features that can directly be extracted from text corpora and the target words themselves by help of computer programs^1^, i.e., no subjective rating data for quantifying lexical features were used as predictors. The machine learning programs (classifiers) were based on *scikit-learn* scripts (Pedregosa et al., 2011). The general procedure was similar to previous research in which we successfully classified verbal materials into motif or author categories or predicted response variables such as word liking and metaphor goodness ratings (Jacobs et al., 2016, 2017; Jacobs and Kinder, 2017, in press).

## Databases and features

The *sdeWaC* corpus (>40 million sentences, ~1 billion word tokens and 6 million types; Baroni et al., 2009) was used for computing reliable lexical indices (e.g., word frequency or orthographic neighborhood density/N), as well as other variables known to influence word recognition (e.g., Jacobs and Grainger, 1994) because its hit rate (overlap between words in database and 300 target words) was high: 74% (211/300). A complication was added by the relatively low hit rate of the German wordnet database (*GermaNet/GN*; Henrich et al., 2012)—crucial for computing word similarity based on semantic relatedness: 43%. Thus, overall 130 target words remained for final analysis [75 beautiful and 55 ugly ones; see S1 in Appendix (Supplementary Material)].

Anecdotal evidence (Limbach, 2004) and results from previous research (Jacobs et al., 2016) suggest that the liking and subjective beauty of even such simple verbal materials as single words can depend on quantifiable features in about all of the 16 cells of the 4 × 4 QNA matrix proposed in Jacobs (2015b). Thus, in the above mentioned book on the most beautiful German words, a 9-year old boy explains why the German word LIBELLE (dragonfly) is the most beautiful for him by referring to features at the sublexical *phonological* level (e.g., the three “Ls” which make the word glide so well on the tongue), or the lexical, *affective-semantic* level (e.g., he loves seeing dragonflies wobble and finds that the word expresses this feeling, that it ensures that one is not afraid of these insects).

Given that more than 50 word features could already be quantified a decade ago for monosyllabic 4–6 letter words (e.g., Graf et al., 2005), a central issue in this field is to investigate which of the myriad of features are distinctive or potentially relevant in the aesthetic appreciation of poetry (cf. Knoop et al., 2016). Exploratory predictive modeling can help identify such features from a large candidate set (Jacobs et al., 2016, 2017; Jacobs and Kinder, 2017, in press). The present approach is basically an exploratory one using a limited set of features that can easily be computed from *sdeWaC* and *GN* or similar corpora for any given target at hand without recurring to standard rating-based word lists like the BAWL (whose hit rate for the present targets was far too low to be useful). Given these constraints and based on the results of pilot studies looking at potentially relevant predictors of the beauty ratings from Jacobs et al. (2015), I selected the following eight tentative features, also in an attempt to keep things as simple as possible and to facilitate follow-up studies, especially of the experimental kind (complementing the present computational one)^2^. The two sublexical (i.e., syllable-based) features were *number of syllables* and *sonority score* (cf. Jacobs and Kinder, in press; see Appendix A in Supplementary Material). The six lexical features were *word length* (number of letters), *surprisal* (-log2 of sdewac-based word frequency), *orthographic neighborhood density* (N), *word similarity* (i.e., GN-based semantic relatedness between all 130 target words), *valence* (parametric positivity/negativity value), and *aesthetic potential* (AP; see Appendix B in Supplementary Material).

## Classifier study and results

**Figure 1:**
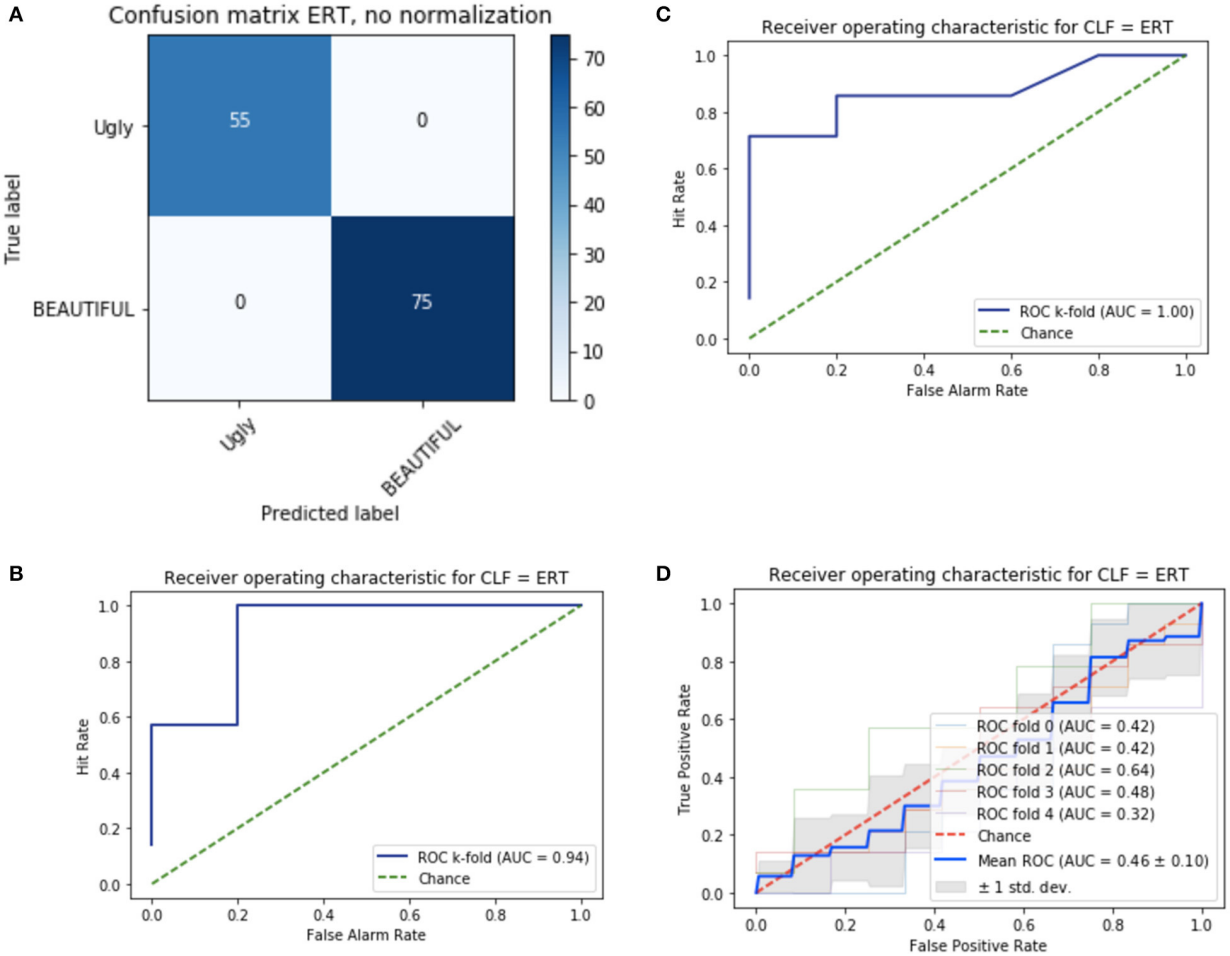
Confusion matrix **(A)** and Receiver Operating Characteristic (ROC, **B–D**) for the ERT classifier (CLF) with eight input variables; **(B)** original data set with parameters set 1; **(C)** original data set with parameter set 2 (see Appendix C in Supplementary Material for details); **(D)** permuted data set. **(D)** Shows the ROCs for five consecutive runs of the k-fold cross-validation for the randomized data set which were all at chance level.

Each target was transformed into a vector based on the eight features, then used as input for machine learning tools classifying each word into one of two categories. Based on successful previous applications (cf. Jacobs and Kinder, 2017, in press), I used the ERT classifier to predict binary categorical ratings (i.e., beautiful vs. ugly; see Appendix C in Supplementary Material). As shown in Figure 1A, the performance of the classifier when training and test set were identical, as assessed by a confusion matrix, is flawless. When using the stratified k-fold cross validation method for evaluating the classifier's predictive performance (prediction of test data on basis of training data), the classifier's performance was excellent with parameter set 1 and perfect with parameter set 2 (Area Under Curve/AUC = 0.94 and 1.0, respectively; see Figures 1B,C and Appendix C in Supplementary Material). As an additional check against overfitting, I applied a second model evaluation technique. Using a permutation test I checked that the classifier's performance was around chance level (AUC = 0.5) when the labels “beautiful vs. ugly” were randomly attributed to the 130 target words (see Figure 1D).

The ERT classifier allows an estimation of the feature importances (which can be interpreted as a descriptive ranking of the predictor variables, Strobl et al., 2009). This ranking suggests that one out of the eight features was of minor importance for the classifier's performance (importance <0.1: N), while *word length* (in letters and number of syllables) and *AP* (all >0.15) appear to be vital predictors, followed by *sonority score* and *surprisal* (0.12), as well as *word similarity* and *valence* (0.11)^3^.

## Discussion

The results show that a potent classifier fed with eight input features can excellently predict whether a German word from the present database is judged as beautiful or ugly, generalizing perfectly from a training to a test data set. Two predictors seem crucial for the classification at hand: a surface feature (word length) and a semantic one (AP). The AP is a novel feature introduced in this paper specifically for assessing the aesthetic potential of words. In a one-way ANOVA, AP was significantly higher for beautiful than for ugly words [*z*-values: 0.25 vs. −0.33; *F*_(1, 128)_ = 12.06, *p* < 0.0007, *R*^2^adj. = 0.08], although the effect of this feature alone is very small. Still, its success as an important predictor of word beauty –in concert with seven others– is first validating evidence for the proposed list of 124 labels and should motivate future use in studies on reading literature. The number of syllables as crucial predictor is notable, since –much like number of letters– its mean value did not differ significantly between the two word groups and it was not strongly correlated with number of letters (*R*^2^ < 0.57). Still, nonlinear, nonparametric supervised learning methods like *decision trees* can produce results largely differing from linear analyses due to their power of detecting hidden structure in complex data sets, e.g., by recursively scanning and (re-)combining variables (LeCun et al., 2015), and of dealing with complex interactions that are difficult to model in a mixed-effects logistic framework (Tagliamonte and Baayen, 2012).

The sonority score is a sublexical feature estimating the phonological aesthetic potential of words and phrases (Jacobs and Kinder, in press). Poetic language expertly plays with the sound-meaning nexus (Schrott and Jacobs, 2011; Aryani et al., 2013, 2016; Schmidtke et al., 2014b; Jacobs, 2015b,c; Jacobs et al., 2015; Ullrich et al., 2017) and thus it would not be surprising that words judged to be more beautiful show higher sonority scores –just as the anecdotal evidence reported above suggests. This was indeed the case [beautiful: 3.12 vs. ugly: 2.9; *F*_(1, 128)_ = 4.6, *p* < 0.033, *R*^2^adj. = 0.03]. Through a process of phonological recoding in silent reading (Ziegler and Jacobs, 1995; Braun et al., 2009) which may play a key role especially in reading poetic texts (Kraxenberger, 2017), the implicit sonority of a written word could more or less unconsciously influence its beauty ratings, a speculation to be tested in future studies.

Surprisal has successfully predicted eye movement or brain wave parameters and correlates positively with reading time (Frank, 2013). Here it also predicted beauty ratings. Regarding word similarity the issue behind the GN-based measure was whether beautiful and ugly words differ in their within-group semantic relatedness. Although the difference was not significant in a linear regression (*p* = 0.83), the classifier makes use of this feature in concert with the other seven as it does with valence. The fact that descriptively valence was not as important as AP may in part be due its computation being based on altogether 36 labels (instead of 124 for AP). Moreover, based on fMRI and EEG results by Briesemeister et al. (2014, 2015) and Kuhlmann et al. (2016), respectively, we proposed that valence itself is a super-feature likely to be derived from core affects like joy or disgust, which is indirectly supported by the present results for the AP feature (Jacobs et al., 2016). Since valence and AP are not correlated (*R*^2^ = 0.005), it could be used in its own right in future studies interested in affective lexical semantics (e.g., Sylvester et al., 2016) rather than aesthetics.

In sum, while none of the eight features on its own accounts for much variance in the data, when processed by the ERT classifier, they seem to fit almost perfectly together in predicting word beauty and perhaps reflect what Kintsch (2012) called *harmony* –how well parts fit the whole. Thus, if a German word features an optimal length (in this corpus: about 12 letters), a specific combination of sonorous syllables, semantic associations with words like ANMUT (grace) or FREUDE (joy) and is rather surprising, it has an increased likelihood of being classified as beautiful. If these features fit together well and, additionally, also with the object that the word denotes, e.g., an aurora, then the word is likely beautiful. While other aspects not considered in the present analyses may also play a role (e.g., arousal, imageability), the present computational eight-feature model of word beauty can serve as a “null-model” against which to test more sophisticated future process models.

## Some predictions for neurocognitive poetics

Each of the eight features can, in principle, be used as a parametric regressor in fMRI studies on literary materials, e.g., to investigate whether similar neural networks in the ventral striatum and medial prefrontal cortex that were associated with beauty ratings of German proverbs (Bohrn et al., 2013) also are responsive to at least some of the present eight features, in particular the AP. It would also be interesting to run an fMRI decoding study (e.g., Haynes, 2015) in which the present ERT classifier is used to predict whether a word was beautiful or ugly on the basis of the participants' brain activity patterns and where the present feature importances could be compared with estimates of neuronal variable importance (e.g., Oh et al., 2003). As concerns the more general issues^4^ (i) to what extent beauty ratings reflect the beauty of the words and/or that of their referents, and (ii) whether similar results can be obtained in other languages (e.g., French or Chinese), future cross-cultural neuroimaging studies could address a question raised previously (Jacobs et al., 2016): to what extent an AP value is computed in the brain from (1) neural activation patterns distributed over the sensory-motor representations of a word's referents (*experiential* aspect) and (2) the size and density of their context (*distributional* aspect), as computationally modeled using co-occurrence statistics, for example (Hofmann and Jacobs, 2014).

## Author contributions

The author confirms being the sole contributor of this work and approved it for publication.

### Conflict of interest statement

The author declares that the research was conducted in the absence of any commercial or financial relationships that could be construed as a potential conflict of interest.

## Footnotes

^1^Python scripts developed by the author.

^2^I am aware of potential shortcomings of such a (non-exhaustive) QNA-based feature set and have discussed them extensively elsewhere (see limitations and outlook sections in Jacobs and Kinder, 2017; Jacobs et al., 2017). Still, I know of no other study that proposes an alternative set and would like to motivate just this: further research attempting to identify distinctive features driving the beauty of words and figurative language in general (cf. Jacobs, 2015b,c).

^3^Note that this ranking can vary with repeated runs and alternative parameter sets of the classifier due to its probabilistic nature. The values reported here are mean values averaged across 10 repetitive runs with parameter set 2 which yielded optimal performance (see Appendix C in Supplementary Material). Of course, much as for multiple linear regressions, adding or deleting features to the list may alter this ranking.

^4^I thank reviewer 2 for this suggestion.
