# Corrigendum: Quantifying the Beauty of Words: A Neurocognitive Poetics Perspective

**DOI:** 10.3389/fnhum.2018.00012

**Published:** 2018-01-30

**Authors:** Arthur M. Jacobs

**Affiliations:** ^1^Department of Experimental and Neurocognitive Psychology, Freie Universität Berlin, Berlin, Germany; ^2^Dahlem Institute for Neuroimaging of Emotion, Berlin, Germany; ^3^Center for Cognitive Neuroscience Berlin, Berlin, Germany

**Keywords:** neurocognitive poetics, quantitative narrative analysis, machine learning, digital humanities, neuroaesthetics, computational stylistics, literary reading, decision trees

In the original article, Equation (1) in Appendix B in Data Sheet 1 contains an error. The correct equation is:

(1) mean[GNsim(*word*, label_1pos) + … + GNsim(*word*, label_Npos)] − mean[GNsim(*word*, label_1neg) + … + GNsim(*word*, label_Nneg)]

where GNsim is the so-called Lin similarity (Lin, 1998) defining semantic relatedness via a formula derived from information theory. This measure is sometimes called a universal semantic similarity measure as it is supposed to be application-, domain-, and resource independent (cf. Budanitsky and Hirst, 2006).

label_1pos and label_1neg/label_Npos and label_Nneg are the first and last terms, respectively, in either the valence or AP lists given in S2 and S3 of the supplementary materials, i.e., BEFRIEDIGUNG (satisfaction), ANGST (fear), or VERGNÜGEN (have fun), TRAUERN (mourn), and ANMUT (grace), WONNE (delight), or ABSCHEU (abomination), ZUMUTUNG (impertinence).

The original file Data Sheet 1 in the Supplementary Material has been updated.

## Conflict of interest statement

The author declares that the research was conducted in the absence of any commercial or financial relationships that could be construed as a potential conflict of interest.
