# Supplementary material for: Quantifying the Beauty of Words: A Neurocognitive Poetics Perspective
Source: Front Hum Neurosci. 2017 Dec 19;11:622. doi: 10.3389/fnhum.2017.00622 (PMC5742167; doi:10.3389/fnhum.2017.00622)
Supplement: Supplementary file 1 [file DataSheet1.docx]

Appendix

**A Computing the sonority score**

Following previous work (Jacobs and Kinder, 2018; Stenneken et al., 2005) and considering that here we deal with written instead of spoken words, I used a simplified index inspired by the sonority hierarchy of German phonemes which yields seven ranks (cf. Vennemann, 1988). The ranks were the following (from highest to lowest): [a] > [e o] > [i u] > [j w y] > [l r] > [m n] > [b c d f g h k p q s t v x z]. Each word in both groups was assigned a value according to the number of letters belonging to the seven rank sets. To control for word length, the sum of the values was divided by number of letters. Thus AUGENWEIDE (eye candy) would get a value of 1*7[a] + 3*6[e o] + 2*5 [i u] + 1*4 [w] + 1*2[n] + 2*1 [d g] = 43/10 = 4.3, whereas NICHTSNUTZ (no-good) would get a value of 2.

**B Computing word similarity, valence and AP**

Following upon an early unsupervised learning approach proposed by Turney and Littman (2003) and own previous research (Westbury et al., 2014), I computed the lexical features *valence* and *aesthetic* *potential* (AP) on the basis of (taxonomy-based) semantic associations of a target word with a set of *labels*, i.e. key words assumed to be prototypical for a certain affect. I chose the term AP to indicate that the label list potentially captures a special aspect of semantic relatedness which goes beyond simple valence (i.e., positivity vs. negativity) and has to do with the aesthetic space of literature, as e.g. investigated in the study by Knoop et al. (2016) –which found adjectives similar to some of those used in the list in S3– and also to express the tentativeness of the concept and method (i.e., still lacking a model of single word beauty, the labels are pretty much ad hoc or intuitive, inspired only by our previous model-guided labels for valence and arousal). However, given the special nature of the present 130 target words, the standard labels used in *sentiment analyses* were suboptimal for several reasons. First, I needed a list that includes terms related to beauty and ugliness. Second, given that the present 130 target words include nouns, verbs and adjectives, the noun list we successfully had used before (set 2 in Table 2 of Westbury et al., 2014) was also suboptimal. Third, it was challenging to find a novel set of labels which would optimize the task at hand, i.e. helping to successfully classify a given list into beautiful vs. ugly words. After extensive pilot studies, I came up with a tentative novel list of 62 positive and 62 negative words for computing the AP for each target word. Still, as a simpler ‘control model’ for the novel list, I adapted the Westbury et al. (2014) *set 2* list to the present purpose for computing valence values for each word (see supplementary materials S2 and S3).

The procedure for computing valence and AP –implemented as a python script based on the pygermanet package freely avalaible on GitHub (Copyright (c) 23 March, 2014 Will Roberts <[wildwilhelm@gmail.com](mailto:wildwilhelm@gmail.com)>)– was as follows. The script compared every target word with every label word in the GN database and computed the pairwise similarities (GNsim in equation 1 below), summed and averaged them for each target word and then computed the difference between the mean for the positive and negative AP label lists.

1. mean[GNsim(*word*, label_1pos) + ... + GNsim(*word*, label_Npos)] – mean[GNsim(*word*, label_1neg) + ... + GNsim(*word*, label_Nneg)]

where GNsim is the so-called Lin similarity (Lin, 1998) defining semantic relatedness via a formula derived from information theory. This measure is sometimes called a universal semantic similarity measure as it is supposed to be application-, domain-, and resource independent (cf. Budanitzky & Hirst, 2006).

*label_1pos* and *label_1neg/* *label_Npos* and *label_Nneg* are the first and last terms, respectively, in either the valence or AP lists given in S2 and S3 of the supplementary materials, i.e., BEFRIEDIGUNG (satisfaction), ANGST (fear), or VERGNÜGEN (have fun), TRAUERN (mourn), and ANMUT (grace), WONNE (delight), or ABSCHEU (abomination), ZUMUTUNG (impertinence).

**C Classifiers and Scores for QNA-based modeling (eight input features)**

Extensive pilot studies using different classifiers (e.g., support vector machines, stochastic gradient descent, simple decision trees) and cross-validation methods (e.g., leave one out, shuffle-split) as well as previous work (Jacobs & Kinder, 2018) indicated the Extremely Random Tree (ERT) as the most powerful for the present selective QNA data set (i.e., sample size, number of features). *Stratified* k-fold cross validation is used for evaluating the classifier’s predictive performance (prediction of test data set on basis of training data set; k = 10 with the following standard training and test sets implemented in *sklearn*: 5 x 116 variable training items vs. 14 variable test items and 5 x 118 training items vs. 12 test items). Parameter set 1 was: nbr trees in forest = 5, minimum number of samples required to split an internal node = 5; parameter set 2 was: trees in forest = 30, minimum number of samples = 2)^[[1]](#footnote-1)^.

The following scores for six alternative classifiers from the sklearn python package were obtained. Scores are given for non-cross-validated runs, i.e. training and test sets were identical (for comparison: ERT score = 1.0 with number of trees = 30; minimum samples per split = 2).

Naive Bayes: 0.646

Gauss Naive Bayes: 0.738

Stochastic Gradient: 0.669

Random Forest: 0.992

ADA boost: 0.885

Decision Tree: 0.884

1. A parameter variation was suggested by a reviewer and, as can be expected, it influenced the classifier’s performance, although not so much. More systematic parameter variations and competitive classifier evaluations are beyond the scope of this explorative perspective paper, though. [↑](#footnote-ref-1)
