# Supplementary material for: Quantifying the Beauty of Words: A Neurocognitive Poetics Perspective
Source: Front Hum Neurosci. 2017 Dec 19;11:622. doi: 10.3389/fnhum.2017.00622 (PMC5742167; doi:10.3389/fnhum.2017.00622)
Supplement: Supplementary file 2 [file DataSheet2.docx]

**Data sheet supplementary materials**

**S1. Target Words (75 beautiful = 1, 55 ugly = 0)**

abscheuerregend 0

Altweibersommer 1

Appetitlosigkeit 0

aquarellieren 1

Arschloch 0

aufheitern 1

Augenweide 1

auserlesen 1

Aussichtslosigkeit 0

Bedeutungslosigkeit 0

Behutsamkeit 1

blutunterlaufen 0

Bordsteinschwalbe 0

Brustschwimmen 1

buntschillernd 1

einschlummern 1

Eiterbeule 0

Ekzem 0

elfenbeinfarben 1

Erbarmungslosigkeit 0

Erfahrungsschatz 1

feindschaftlich 0

ficken 0

Fotze 0

Friedfertigkeit 1

frohlocken 1

Geborgenheit 1

Geistesblitz 1

Gewissensfrage 1

Giftmischer 0

Habseligkeit 1

Hausschlachtung 0

Henkersmahlzeit 0

Herbstzeitlose 0

hospitalisieren 0

Hurensohn 0

Infektionskrankheit 0

kacken 0

Kapitalverbrechen 0

Kichererbse 1

kinderfeindlich 0

Kinkerlitzchen 1

kotzen 0

krankheitserregend 0

Kriegsdienst 0

Kriegsgefangenschaft 0

Kuddelmuddel 1

Kulturbeutel 1

Lautmalerei 1

Lebenslust 1

Libelle 1

Liebesbeziehung 1

Liebeskummer 1

Lieblingsspeise 1

liebreizend 1

Luftikus 0

Minderwertigkeit 0

Miniaturausgabe 1

Minnesang 1

Missgeburt 0

Mittsommernacht 1

Mondschein 1

Nervenheilanstalt 0

Nichtsnutz 0

Nutte 0

Pampelmuse 1

Pantoffeltierchen 1

Pickel 0

Quentchen 1

Rassendiskriminierung 0

Rechenschieber 1

Regenbogen 1

Regenbogenforelle 1

Rodelschlitten 1

Schabernack 1

Schafskopf 0

Schattenspiel 1

schimmeln 0

schlachten 0

schlaftrunken 1

Schlaraffenland 1

Schmerzhaftigkeit 0

schmerzlindernd 1

Schreckensnachricht 0

Schurkenstaat 0

Schwuchtel 0

Seelenverwandtschaft 1

sehnsuchtsvoll 1

Seifenblase 1

Silberfischchen 1

Sinneswandel 1

Sittlichkeitsverbrechen 0

Situationskomik 1

Sommerfrische 1

Sommerregen 1

sonnendurchflutet 1

Sonnenstrahl 1

Sonntagskind 1

Spiegelschrift 1

Staatssicherheitsdienst 0

Steckenpferd 1

Stelldichein 1

Sternenstaub 1

Sternschnuppe 1

Steuerhinterziehung 0

Stinkefinger 0

Streicheleinheit 1

Tagtraum 1

Tausendsassa 1

Techtelmechtel 1

Terrorangriff 0

Todesstrafe 0

Trunkenbold 0

Unbarmherzigkeit 0

unterprivilegiert 0

vergewaltigen 0

Vergissmeinnicht 1

verpesten 0

verrotten 0

Warze 0

Weltwirtschaftskrise 0

wichsen 0

Willensfreiheit 1

wirklichkeitsfremd 0

wissensdurstig 1

Zauberformel 1

Zitronenfalter 1

Zitronenpresse 1

zusammenbleiben 1

Zweisamkeit 1

**S2. Valence Lists (18 positive and 18 negative labels; cf. Westbury et al., 2014, Table 2, row 2)**

positive = ['Befriedigung','Erleichterung','Freude','Glück','Zufriedenheit',’Stolz’,’Überraschung’,'befriedigt','erleichtert','freudig', 'glücklich','zufrieden',’stolz’,'befriedigen','erleichtern','freuen',’erfreuen’,’beglücken’]

negative = ['Angst',’Furcht’,'Ekel','Scham','Trauer','Verlegenheit','verängstigt',’furchtsam’,'ekelig','beschämt','traurig','verlegen’,'ängstigen','ekeln','schämen',’beschämen’,'trauern']

**S3. Aesthetic potential (AP) Lists (62 positive and 62 negative labels)**

pos_labels = ['Anmut',

'anmutig',

'anziehen',

'anziehend',

'begeistert',

'Begeisterung',

'beschützen',

'bewegend',

'erheitern',

'erfreuen',

'exzellent',

'Faszination',

'faszinieren',

'Einheit',

'Entzücken',

'Freude',

'Genuss',

'gesund',

'Gesundheit',

'Glück',

'glücklich',

'Güte',

'gut',

'herrlich',

'Herz',

'heilend',

'Heiterkeit',

'hoffen',

'Hoffnung',

'hübsch',

'Kultur',

'Kunst',

'Liebe',

'lieben',

'liebevoll',

'lieblich',

'malerisch',

'Musik',

'musizieren',

'Paradies',

'paradiesisch',

'Perfektion',

'perfekt',

'prächtig',

'Poesie',

'poetisch',

'Reichtum',

'reich',

'rührend',

'Rührung',

'schön',

'Schönheit',

'Schutz',

'Sommer',

'sommerlich',

'Sonne',

'sonnig',

'Stil',

'stilecht',

'Überraschung',

'Vergnügen',

'Wonne']

neg_labels = ['Abscheu',

'abscheulich',

'abschrecken',

'Ärger',

'Angst',

'anekeln',

'ankotzen',

'arm',

'Armseligkeit',

'Armut',

'Billigkeit',

'böse',

'boshaft',

'Betrug',

'chaotisch',

'Ekel',

'ekeln',

'fies',

'grässlich',

'Grauen',

'grauenhaft',

'hässlich',

'Hässlichkeit',

'hassen',

'Horror',

'Irre',

'Kitsch',

'kitschig',

'Krach',

'Krankheit',

'Lump',

'Misere',

'Missbrauch',

'missbrauchen',

'misslich',

'Mord',

'pissen',

'prügeln',

'Ramsch',

'Scham',

'schlecht',

'Schmerz',

'schmerzen',

'Schund',

'stinken',

'Strafe',

'Streit',

'tödlich',

'Tod',

'töten',

'übel',

'Übelkeit',

'Unglück',

'Unkultur',

'unschön',

'verderben',

'Verlust',

'verachten',

'versagen',

'verzweifeln',

'Wut',

'Zumutung']
